# Supplementary material for: Outcome differences by sex in oncology clinical trials
Source: Nat Commun. 2024 Mar 23;15:2608. doi: 10.1038/s41467-024-46945-x (PMC10960820; doi:10.1038/s41467-024-46945-x)
Supplement: Supplementary file 1 — Supplementary Information [file 41467_2024_46945_MOESM1_ESM.pdf]

Outcome differences by sex in oncology clinical trials

Supplementary Information

Ashwin V. Kammula B.S.[1], Alejandro A. Schäffer Ph.D.[1*], Padma Sheila Rajagopal M.D., M.P.H., M.Sc.[1,2], Razelle Kurzrock M.D.[3], Eytan Ruppin M.D., Ph.D.[1*]

[1] Cancer Data Science Laboratory, Center for Cancer Research, National Cancer Institute, Bethesda, MD 20892 USA

[2] Women's Malignancies Branch, Center for Cancer Research, National Cancer Institute, Bethesda, MD 20892 USA

[3] WIN Consortium and Medical College of Wisconsin, Milwaukee, WI 53226 and University of Nebraska, Omaha, NE 68198 USA

[*] Corresponding authors

Correspondence to:

Alejandro A. Schäffer or Eytan Ruppin

Building 15-C1

CDSL/NCI/NIH

Bethesda, MD, 20892, USA

E-mail: alejandro.schaffer@nih.gov or eytan.ruppin@nih.gov

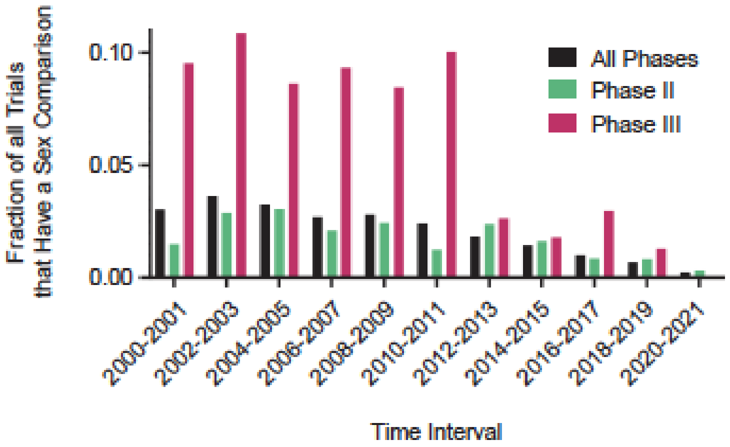

Supplementary Figure 1. Sex comparisons over time by trial phase. Candidate trials are defined by passing our initial filtering of having results, patients of both sexes, and more than twenty-five patients enrolled (or not reported). Black: All candidate trials were placed into bins of two years spanning from 2000 to 2021. The fraction of trials with found sex comparisons of total candidate trials was calculated for each bin. Green: The fraction of Phase II sex comparison trials of Phase II candidate trials for each bin. Pink: The fraction of Phase III sex comparison trials of Phase III candidate trials for each bin. In each group, we observe a decrease in the fraction of sex comparison trials over time. The source data for this figure are provided in Supplementary Table 3 and equivalently in the Source Data file.

**Category**	**Number of Comparisons**
Similarity	176
Difference - Favors Males	127
Difference - Favors Females	221
Difference - Favors Unknown	8
**Total**	**532**

Supplementary Table 1. Counts of sex comparisons in post-treatment outcomes or side effects according to which sex is favored. The comparisons for which the favored sex is unknown come mostly from meeting abstracts that are written with key details lacking.

Drug Category	Male	Female	Same	Favors	P-value	FDR
Targeted	12	43	43	Female	1.21772E-05	8.52404E-05
Chemo	16	38	42	Female	0.001171003	0.004098512
Antibody	7	21	10	Female	0.004410893	0.010292083
Other	2	9	7	Female	0.026855469	0.04699707
Immunotherapy	6	3	6	Male	0.1640625	0.2296875
Antibody-drug conjugate	1	3	2	Female	0.25	0.291666667
Immune-Other	2	3	5	Female	0.3125	0.3125
Supportive	1	2	3	Female	ND	
**Total**	**47**	**122**	**118**			

Supplementary Table 2. Counts of trials with multivariate or univariate analysis of SOR according to the highest drug category used among eight categories and according to which sex had the better SOR. In this Table each trial was counted at most once. There was one 'Same' trial excluded from this table because the drug used was unclear. The p-values are for a two-sided binomial test that the values in the "Male" and "Female" columns are in 1:1 proportion. The false discovery rate (FDR) is a corrected p-value using the Benjamini-Hochberg method. The p-values were only calculated if the sum of the values in the "Male" and "Female" columns is at least four. If the "Male" and "Female" columns contain fewer than four trials total, the "P-value" has the value ND (Not Done), and "FDR" is blank.

Years	Sex Comp. Trials	Eligible Trials	Fraction	Phase II Fraction	Phase III Fraction
2000-2001	29	952	0.03064	0.015009	0.095238
2002-2003	47	1297	0.03624	0.028834	0.108696
2004-2005	58	1789	0.03242	0.030602	0.086364
2006-2007	54	2002	0.02697	0.021005	0.093385
2008-2009	58	2088	0.02805	0.024299	0.084677
2010-2011	49	2037	0.02406	0.012405	0.100418
2012-2013	34	1857	0.01831	0.02381	0.026415
2014-2015	28	1937	0.01446	0.016311	0.017986
2016-2017	20	1985	0.01008	0.008454	0.029787
2018-2019	12	1726	0.00695	0.008152	0.012821
2020-2021	2	801	0.0025	0.003311	0

Supplementary Table 3. Data on number of trials that have a sex comparison according to the starting two-year interval and the trial phase. “Sex Comp. Trials” are trials which were found to have a sex comparison. “Eligible Trials” are trials which have results, patients of both sexes, and more than twenty-five patients enrolled (or not reported). “Fraction” is the ratio of “Sex Comp. Trials” to “Eligible Trials.” “Phase II Fraction” and “Phase III Fraction” is the ratio of Sex Comp Trials to Eligible Trials when filtering to only trials from Phase II or Phase III, respectively. This data are the source data for Supplementary Figure 1.

Start Year	Eligible Trials	Trials with a Sex Comparison	Proportion
1993	19	0	0
1994	37	3	0.08108108
1995	59	3	0.05084746
1996	89	5	0.05617978
1997	109	1	0.00917431
1998	205	7	0.03414634
1999	253	5	0.01976285
2000	323	7	0.02167183
2001	381	11	0.02887139
2002	412	6	0.01456311
2003	484	10	0.02066116
2004	653	15	0.0229709
2005	705	11	0.01560284
2006	782	12	0.01534527
2007	710	14	0.01971831
2008	707	13	0.01838755
2009	687	17	0.02474527
2010	702	15	0.02136752
2011	657	10	0.0152207
2012	632	11	0.01740506
2013	620	7	0.01129032
2014	737	10	0.01356852
2015	822	10	0.01216545
2016	781	5	0.00640205
2017	919	4	0.00435256
2018	957	1	0.00104493
2019	974	2	0.00205339
2020	1064	0	0
2021	1233	0	0
2022	1275	0	0

Supplementary Table 4. Counts of trials that have a known start year during 1993-2022, enrolled more than twenty-five patients, enrolled both males, and females, and had at least one enrollment site in the United States, including Puerto Rico. These requirements are an approximation to the set of trials for which United States Public Health Service Act sec. 492B, 42 U.S.C. sec. 289a-2 required a comparison between males and females to be done.

Disease	Male	Female	Same	Favors	P-value	FDR
Oncology: Lymphoma, Non-Hodgkin's	4	20	9	Female	0.000633	0.005141
Oncology: Lung, Non-Small Cell	13	35	34	Female	0.000685	0.005141
Oncology: Leukemia, Acute Myelogenous	7	15	4	Female	0.040661	0.203304
Oncology: Renal	0	4	4	Female	0.0625	0.234375
Oncology: Leukemia, Chronic Lymphocytic	2	6	4	Female	0.109375	0.292969
Oncology: Colorectal	3	7	15	Female	0.117188	0.292969
Oncology: Gastric	5	2	2	Male	0.164063	0.315505
Oncology: Leukemia, Chronic Myelogenous	4	2	5	Male	0.234375	0.315505
Oncology: Lung, Small Cell	4	2	5	Male	0.234375	0.315505
Oncology: Myelodysplastic Syndrome	5	4	3	Male	0.246094	0.315505
Oncology: Liver	3	1	3	Male	0.25	0.315505
Oncology: Melanoma	4	4	5	Neither	0.273438	0.315505
Oncology: Leukemia, Acute Lymphocytic	3	4	6	Female	0.273438	0.315505
Oncology: (N/A)	2	2	0	Neither	0.375	0.375
Oncology: Esophageal	2	2	1	Neither	0.375	0.375
Metabolic/Endocrinology: Anemia	0	1	0	Female	ND	
Metabolic/Endocrinology: Metachromatic Leukodystrophy (MLD)	1	0	0	Male	ND	
Oncology: Anal	1	2	0	Female	ND	
Oncology: Bile Duct (Cholangiocarcinoma)	0	2	0	Female	ND	
Oncology: Bladder	0	2	2	Female	ND	
Oncology: Breast	0	1	1	Female	ND	
Oncology: CNS, Glioblastoma	0	1	0	Female	ND	
Oncology: Endometrial	1	0	0	Male	ND	
Oncology: GIST	0	2	2	Female	ND	
Oncology: Gallbladder	0	2	0	Female	ND	
Oncology: Head/Neck	2	0	5	Male	ND	
Oncology: Lymphoma, Hodgkin's	1	0	4	Male	ND	
Oncology: Mesothelioma	0	2	1	Female	ND	
Oncology: Multiple Myeloma	0	3	7	Female	ND	
Oncology: Myeloproliferative Neoplasms	1	2	2	Female	ND	
Oncology: Neuroblastoma	1	0	0	Male	ND	
Oncology: Neuroendocrine	2	1	5	Male	ND	
Oncology: Pancreas	0	3	3	Female	ND	
Oncology: Skin, Basal Cell Carcinoma	0	1	0	Female	ND	
Oncology: Soft Tissue Sarcoma	0	2	2	Female	ND	
Oncology: Supportive Care	2	1	4	Male	ND	
Oncology: Unspecified Cancer	0	1	1	Female	ND	

Supplementary Table 5. Each row is for one malignancy, described in the “Disease” column, which was used in at least one trial that has a sex comparison for survival, outcome, or response (SOR) that uses multivariate or univariate analysis. The “Male” and Female” columns count how many trials favored males and females, respectively. The “Same” column counts how many trials had no difference between males and females. The “Favors” columns indicates with one word whether Males, Females, or Neither is in the majority. The p-values are for a two-sided binomial test that the values in the “Male” and “Female” columns are in 1:1 proportion. The false discovery rate (FDR) is a corrected p-value using the Benjamini-Hochberg method. The p-values were only calculated if the sum of the values in the “Male” and “Female” columns is at least four. If the “Male” and “Female” columns contain fewer than four trials total, the “P-value” has the value ND (Not Done), and “FDR” is blank.

Treatment	Male	Female	Same	Total	Favors	P-value	FDR
erlotinib	0	10	5	15	Female	0.000976563	0.00390625
gefitinib	0	9	7	16	Female	0.001953125	0.00390625
cisplatin	2	3	2	7	Female	0.3125	0.375
carboplatin (iv)	2	2	2	6	Neither	0.375	0.375
gemcitabine hydrochloride	0	3	5	8	Female	ND	
paclitaxel	0	2	3	5	Female	ND	
docetaxel	0	3	2	5	Female	ND	
pemetrexed disodium	0	2	2	4	Female	ND	
vinorelbine	2	1	1	4	Male	ND	
bevacizumab	1	1	1	3	Neither	ND	
nivolumab	1	2	0	3	Female	ND	
TS-1	0	0	2	2	Neither	ND	
afatinib	0	1	1	2	Female	ND	
vandetanib	1	0	1	2	Male	ND	
veliparib	2	0	0	2	Male	ND	
Pulmicort	0	0	1	1	Neither	ND	
TSPP vaccine	0	0	1	1	Neither	ND	
amrubicin (IV)	0	0	1	1	Neither	ND	
crizotinib (tablet)	0	0	1	1	Neither	ND	
odetiglucan	0	0	1	1	Neither	ND	
ponatinib	0	0	1	1	Neither	ND	
sorafenib	0	0	1	1	Neither	ND	
surgical intervention	0	0	1	1	Neither	ND	
vadimezan	0	0	1	1	Neither	ND	
aprepitant (capsule)	1	0	0	1	Male	ND	
atezolizumab	0	1	0	1	Female	ND	
camrelizumab	1	0	0	1	Male	ND	
cetuximab	0	1	0	1	Female	ND	
dexamethasone	1	0	0	1	Male	ND	
etoposide	1	0	0	1	Male	ND	
granisetron	1	0	0	1	Male	ND	
irinotecan (IV)	0	1	0	1	Female	ND	
onartuzumab	0	1	0	1	Female	ND	
palonosetron (IV)	1	0	0	1	Male	ND	
palonosetron (oral)	1	0	0	1	Male	ND	
pelareorep	0	1	0	1	Female	ND	
radiation therapy	1	0	0	1	Male	ND	
tegafur + uracil, Taiho	1	0	0	1	Male	ND	
vinblastine	1	0	0	1	Male	ND	
vindesine	1	0	0	1	Male	ND	
vinorelbine, oral	1	0	0	1	Male	ND	

Supplementary Table 6. Treatments used in non-small cell lung cancer trials with multivariate or univariate SOR analysis according to which sex had better SOR. A trial may be counted for more than one treatment. The p-values are for a two-sided binomial test that the values in the “Male” and “Female” columns are in 1:1 proportion. The false discovery rate (FDR) is a corrected p-value using the Benjamini-Hochberg method. The p-values were only calculated if the sum of the values in the “Male” and “Female” columns is at least four. If the “Male” and “Female” columns contain fewer than four trials total, the “P-value” has the value ND (Not Done), and “FDR” is blank.

Region	Male EGFRi	Male Non-EGFRi	Female EGFRi	Female Non-EGFRi	Same EGFRi	Same Non-EGFRi
Asia	0	7	10	6	5	7
Australia/Oceania	0	1	1	1	1	2
Africa	0	2	1	1	0	1
Americas	0	4	7	5	6	9
Europe	1	4	8	6	8	9

Supplementary Table 7. Reclassification of the non-small cell lung cancer trials with multivariate or univariate SOR analysis according to geographic region of the trial. Trials which utilize EGFR inhibitors are separated from trials using non-EGFR inhibitors. A trial may be counted in more than one region.

Treatment	Male	Female	Same	Total	Favors	P-value	FDR
rituximab	1	10	2	13	Female	0.005371094	0.005371094
fludarabine	0	3	1	4	Female	ND	
lenalidomide	0	1	3	4	Female	ND	
allogeneic stem cells	1	1	1	3	Neither	ND	
cyclophosphamide	0	2	0	2	Female	ND	
cytarabine	0	1	1	2	Female	ND	
doxorubicin	0	2	0	2	Female	ND	
etoposide	0	1	1	2	Female	ND	
methotrexate (IV)	0	1	1	2	Female	ND	
prednisone	0	2	0	2	Female	ND	
vincristine	0	2	0	2	Female	ND	
CD19-CAR T cells, Beijing Doing Biomedical	1	0	0	1	Male	ND	
acalabrutinib	1	0	0	1	Male	ND	
alemtuzumab (SC)	0	1	0	1	Female	ND	
arsenic trioxide, unspecified	0	1	0	1	Female	ND	
asparaginase, pegylated, Enzon	0	0	1	1	Neither	ND	
asparaginase	0	1	0	1	Female	ND	
bendamustine	0	1	0	1	Female	ND	
bortezomib (IV)	0	0	1	1	Neither	ND	
brentuximab vedotin	0	1	0	1	Female	ND	
chlorambucil	0	1	0	1	Female	ND	
daunorubicin	0	1	0	1	Female	ND	
galiximab	0	1	0	1	Female	ND	
hypericin, Hy BioPharma (topical)	0	0	1	1	Neither	ND	
ifosfamide	0	1	0	1	Female	ND	
interferon, Genentech (alpha2a)	0	1	0	1	Female	ND	
melphalan	0	0	1	1	Neither	ND	
mercaptopurine, unspecified	0	1	0	1	Female	ND	
methotrexate (oral)	0	1	0	1	Female	ND	
mitoxantrone	0	1	0	1	Female	ND	
obinutuzumab	0	1	0	1	Female	ND	
treosulfan (IV)	0	0	1	1	Neither	ND	
vitamin C	0	1	0	1	Female	ND	

Supplementary Table 8. Treatments used in non-Hodgkin's lymphoma (NHL) trials with multivariate or univariate SOR analysis according to which sex had better SOR. A trial may be counted for more than

one treatment. The p-values are for a two-sided binomial test that the values in the “Male” and “Female” columns are in 1:1 proportion. The false discovery rate (FDR) is a corrected p-value using the Benjamini-Hochberg method. The p-values were only calculated if the sum of the values in the “Male” and “Female” columns is at least four. If the “Male” and “Female” columns contain fewer than four trials total, the “P-value” has the value ND (Not Done), and “FDR” is blank.

Drug Category	Male	Female	Same	Total	Favors	P-value	FDR
Antibody	4	1	3	8	Male	0.15625	0.273438
Other	4	2	1	7	Male	0.234375	0.273438
Chemo	5	4	4	13	Male	0.246094	0.273438
Targeted	3	4	1	8	Female	0.273438	0.273438
Supportive	3	0	0	3	Male	ND	
Immune-Other	1	1	0	2	Neither	ND	
Immunotherapy	2	0	0	2	Male	ND	
Antibody-drug conjugate	0	0	0	0	Neither	ND	
**Total**	**22**	**12**	**9**	**43**			

Supplementary Table 9. Counts of trials with multivariate or univariate analysis of side effects according to the highest drug category among eight categories and according to which sex had the better SOR. In this Table each trial was counted at most once. There was one 'Female' trial excluded from this table because the drug used was unclear. The p-values are for a two-sided binomial test that the values in the “Male” and “Female” columns are in 1:1 proportion. The false discovery rate (FDR) is a corrected p-value using the Benjamini-Hochberg method. The p-values were only calculated if the sum of the values in the “Male” and “Female” columns is at least four. If the “Male” and “Female” columns contain fewer than four trials total, the “P-value” has the value ND (Not Done), and “FDR” is blank.

Disease	Male	Female	Same	Total	Favors	P-value	FDR
Oncology: Colorectal	10	3	2	15	Male	0.034912	0.069824
Oncology: Multiple Myeloma	2	2	1	5	Neither	0.375	0.375
Oncology: Leukemia, Acute Lymphocytic	1	1	2	4	Neither	ND	
Oncology: Lung, Non-Small Cell	1	1	2	4	Neither	ND	
Oncology: Lymphoma, Non-Hodgkin's	1	1	2	4	Neither	ND	
Oncology: Supportive Care	3	0	1	4	Male	ND	
Oncology: Metastatic Cancer	2	1	0	3	Male	ND	
Oncology: Leukemia, Chronic Myelogenous	1	0	1	2	Male	ND	
Oncology: Lymphoma, Hodgkin's	1	0	1	2	Male	ND	
Oncology: Myelodysplastic Syndrome	0	1	1	2	Female	ND	
Oncology: Unspecified Cancer	1	0	1	2	Male	ND	
CNS: Pain (neuropathic)	1	0	0	1	Male	ND	
Oncology: (N/A)	1	0	0	1	Male	ND	
Oncology: Anal	0	1	0	1	Female	ND	
Oncology: Bladder	1	0	0	1	Male	ND	
Oncology: CNS, Medulloblastoma	1	0	0	1	Male	ND	
Oncology: CNS, Other	1	0	0	1	Male	ND	
Oncology: Esophageal	0	1	0	1	Female	ND	
Oncology: Gastric	0	1	0	1	Female	ND	
Oncology: Lung, Small Cell	1	0	0	1	Male	ND	
Oncology: Melanoma	1	0	0	1	Male	ND	
Oncology: Neuroendocrine	1	0	0	1	Male	ND	
Oncology: Pancreas	0	1	0	1	Female	ND	
Oncology: Renal	1	0	0	1	Male	ND	
Oncology: Soft Tissue Sarcoma	0	1	0	1	Female	ND	
Oncology: Thymus	1	0	0	1	Male	ND	

Supplementary Table 10. Each row is for one malignancy, described in the “Disease” column, which was used in at least one trial that has a sex comparison for side effects that uses multivariate or univariate analysis. The “Male” and “Female” columns count how many trials favored males and females, respectively. The “Same” column counts how many trials had a similarity between males and females. The “Favors” column indicates with one word whether B, C, or Neither is in the majority. The p-values are for a two-sided binomial test that the values in the “Male” and “Female” columns are in 1:1 proportion. The false discovery rate (FDR) is a corrected p-value using the Benjamini-Hochberg method. The p-values were only calculated if the sum of the values in the “Male” and “Female” columns is at least four. If the “Male” and “Female” columns contain fewer than four trials total, the “P-value” has the value ND (Not Done), and “FDR” is blank.

Treatment	Male	Female	Same	Total	Favors	P-value	FDR
oxaliplatin	5	1	0	6	Male	0.09375	0.1875
irinotecan	3	1	0	4	Male	0.25	0.25
bevacizumab	3	0	2	5	Male	ND	
capecitabine	1	1	2	4	Neither	ND	
fluorouracil	3	0	0	3	Male	ND	
leucovorin	3	0	0	3	Male	ND	
MUC1 peptide-poly-ICLC adjuvant vaccine	0	1	0	1	Female	ND	
aprepitant	1	0	0	1	Male	ND	
cetuximab	0	1	0	1	Female	ND	
dexamethasone	1	0	0	1	Male	ND	
fosaprepitant dimeglumine	1	0	0	1	Male	ND	
regorafenib	1	0	0	1	Male	ND	
undisclosed - 5-HT3 antagonist	1	0	0	1	Male	ND	
xaliproden hydrochloride	1	0	0	1	Male	ND	

Supplementary Table 11. Treatments used in colorectal cancer trials with multivariate or univariate analysis side effect comparisons according to which sex had lesser side effects. A trial may be counted for more than one treatment. The p-values are for a two-sided binomial test that the values in the “Male” and “Female” columns are in 1:1 proportion. The false discovery rate (FDR) is a corrected p-value using the Benjamini-Hochberg method. The p-values were only calculated if the sum of the values in the “Male” and “Female” columns is at least four. If the “Male” and “Female” columns contain fewer than four trials total, the “P-value” has the value ND (Not Done), and “FDR” is blank.
