# Supplementary material for: Outcome differences by sex in oncology clinical trials
Source: Nat Commun. 2024 Mar 23;15:2608. doi: 10.1038/s41467-024-46945-x (PMC10960820; doi:10.1038/s41467-024-46945-x)
Supplement: Supplementary file 3 — Description of Additional Supplementary Files [file 41467_2024_46945_MOESM3_ESM.pdf]

Supplementary Data 1. Number of trials with sex comparisons for survival, outcome, or response sorted by treatment. Each row is for one treatment, described in column A, that was used in at least one trial that has a sex comparison for survival, outcome or response (SOR) that uses multivariate or univariate analysis. Columns B and C count how many trials favored Males and Females respectively. Column D counts how many trials had no significant difference between males and females. Column E is the total B+C+D. Column F indicates with one word whether B, C, or Neither is in the majority. Column G is the p-value for a two-sided binomial test of the hypothesis that the values in B and C are in a 1:1 proportion. Column H is the false discovery rate (FDR) corrected p-value using the Benjamini-Hochberg method. The p-values were only calculated if the sum of the values in columns B and C is at least four. If columns B and C contain fewer than four trials total, column G has the value ND (Not Done), and column H is blank.

Supplementary Data 2. Data on number of trials that have a sex comparison according to the start year and phase of each trial. Eligible trials are those which enroll both males and females, have patient accrual > 25 (or with this information unavailable), and have trial results reported. Blank entries in the proportions columns signify that the number of eligible trials is zero.

Supplementary Data 3. Number of trials with sex comparisons for side effects sorted by treatment. Each row is for one treatment, described in column A, that was used in at least one trial that has a sex comparison for any side effects that uses multivariate or univariate analysis. Columns B and C count how many trials favored males and females respectively. Column D counts how many trials had a similarity between males and females. Column E is the total B+C+D. Column F indicates with one word whether B or C, or Neither is in the majority. Column G is the p-value for a two-sided binomial test of the hypothesis that the values in columns B and C are in a 1:1 proportion. Column H is the false-discovery rate (FDR) corrected p-value using the Bejamini-Hochberg Method. The p-values were only calculated if the sum of the values in column B and C is at least 4. If they contain fewer than 4 trials, column G has the value ND (Not Done) and column H is blank.
